# *Sargassum plagiophyllum* Extract Enhances Colonic Functions and Modulates Gut Microbiota in Constipated Mice

**DOI:** 10.3390/nu14030496

**Published:** 2022-01-24

**Authors:** Pissared Khuituan, Nawiya Huipao, Nilobon Jeanmard, Sitthiwach Thantongsakul, Warittha Promjun, Suwarat Chuthong, Chittipong Tipbunjong, Saranya Peerakietkhajorn

**Affiliations:** 1 Division of Health and Applied Sciences, Faculty of Science, Prince of Songkla University, Songkhla 90110, Thailand; pissared.k@psu.ac.th (P.K.); nawiya.h@psu.ac.th (N.H.); chittipong.t@psu.ac.th (C.T.); 2 Gut Biology and Microbiota Research Unit, Prince of Songkla University, Songkhla 90110, Thailand; nilo.jean18@gmail.com (N.J.); 5910210317@psu.ac.th (S.T.); waritthapj@gmail.com (W.P.); suwarat1041@gmail.com (S.C.); 3 Division of Biological Science, Faculty of Science, Prince of Songkla University, Songkhla 90110, Thailand

**Keywords:** brown algae, *Sargassum plagiophyllum*, constipation, gastrointestinal transit, gut microbiota, transepithelial transport

## Abstract

Constipation is a symptom that is widely found in the world’s population. Various dietary supplementations are used to relieve and prevent constipation. Seaweed is widely used for its health benefits. In this study, we aimed to investigate the effects of *Sargassum plagiophyllum* extract (SPE) on functions of the gastrointestinal tract and gut microbiota. The results show that SPE pretreatment increased the frequency of gut contraction, leading to reduce gut transit time. SPE pretreatment also significantly increased the secretion of Cl^−^ and reduced Na^+^ absorption, increasing fecal water content in constipated mice (*p* < 0.05). In addition, the Bifidobacteria population in cecal contents was significantly higher in constipated mice pretreated with 500 mg/kg SPE for 14 days than in untreated constipated mice (*p* < 0.05). Our findings suggest that SPE can prevent constipation in loperamide-induced mice. This study may be useful for the development of human food supplements from *S. plagiophyllum*, which prevent constipation.

## 1. Introduction

Constipation is a health symptom that has been reported to affect approximately 8.2–32.9% of the world’s population [1,2]. Constipation is often defined as infrequent and/or difficult bowel movements with a hard, dry stool [3,4], and it can be brought on by reduced physical activity, insufficient fluid intake, medication, and depression [5]. The condition is associated with gut microbiota imbalances involving decreased numbers of Bifidobacteria and Lactobacilli, increased numbers of pathogens, and suppressed intestinal motility [6]. Bifidobacteria, Lactobacilli, and Enterococci were effectively used in the treatment of constipation [7,8,9], and previous studies revealed that the levels of these bacteria were decreased in irritable bowel syndrome with constipation [10,11]. Several studies also showed that Enterobacteriaceae were increased in the condition of chronic constipation [10,12]. The recommended treatments for constipation include osmotic laxatives, generally lactulose, magnesium oxide, or polyethylene glycol [13], but the overuse of osmotic laxatives can result in dehydration and electrolyte imbalance. Clearly, these laxatives, which are available over the counter, can be harmful if patients incorrectly use them.

Alternative treatments emphasize dietary management to ensure a sufficient intake of dietary fiber and fluids [14,15,16]. Moreover, some nutritional plant products have been reported to aid the management of constipation. The extracts of *Aloe ferox* Mill, agarwood (*Aquilaria sinensis* and *Aquilaria crasna*), *Liriope platyphylla*, and prunes can increase intestinal motility, as well as the frequency and weight of stools. In Japan, the consumption of the seaweed *Ulva prolifera* gives effective relief to constipation sufferers [17]. The nutritional and pharmaceutical benefits of algae have been known for many centuries. Algae contain compounds that exert anti-inflammatory, antimicrobial, and antioxidant effects [18]. They also contain high amounts of dietary fiber, which has been widely used for the treatment of gastrointestinal disorders, including constipation, diarrhea, and ulcerative colitis [17,18,19,20,21]. Usually, the fiber component of algae principally comprises structural polysaccharides. A recent study reported that algal polysaccharides increased the populations of Bifidobacteria and Lactobacilli both in vivo and in vitro [22,23,24]. The large group of brown algae includes the macroalgal genus *Sargassum*, which is widely distributed along the coasts of the Gulf of Thailand and the Andaman Sea [25]. In the *Sargassum* species, the dominant polysaccharides include alginate, laminarin, and fucoidan [26]. The polysaccharides in *Sargassum* have been widely studied in pharmacological research, such as research on anti-obesity, anticancer, anti-inflammatory, antibacterial, and antiviral activities [27,28]. A previous study revealed that the components of *Sargassum plagiophyllum* were 68.69% carbohydrates (including 22.24% fiber), 9.05% protein, 0.88% lipid and 21.38% ash [29]. Fucoidan is a long-chain-sulfated polysaccharide found in *S. plagiophyllum*, which potentially reduces inflammation, and has antioxidant, antitumor, and anti-cholesterol activities [30,31]. Moreover, several studies revealed that *S. plagiophyllum* extract contains phenolic compounds and fucoxanthin, which have therapeutic activity, such as antioxidant, anti-inflammatory, anticancer, anti-obesity, and antidiabetic activities [32,33]. A recent study revealed that *S. plagiophyllum* extract also has antioxidant activity [34].

The present study aimed to investigate the effects of *Sargassum plagiophyllum* extract (SPE) on the changes in colonic functions and gut microbiota in a constipation model of mice. The gut transit time, colonic motility patterns, colonic smooth muscle contractility, electrolyte transport across cell membranes in the colon, and colonic microbiota composition were investigated.

## 2. Materials and Methods

### 2.1. Sargassum plagiophyllum Extract (SPE) Preparation

Adult-stage *Sargassum plagiophyllum* was collected from Lanta Island, Krabi, Thailand. The preparation of SPE followed the method of a previously described extraction of an algal sample [35]. Briefly, 1 g of finely ground dried *S. plagiophyllum* was added to 100 mL of distilled water and autoclaved at 121 °C for 20 min. The autoclaved *S. plagiophyllum* was centrifuged at 2220× *g* for 10 min, and the supernatant was collected and freeze dried to obtain SPE powder.

### 2.2. Animals and Experimental Design

Adult male ICR/Mlac mice (4–5 weeks old, 25–30 g) were obtained from the National Laboratory Animal Center, Mahidol University, Thailand. The mice were reared in a humidity- and temperature-controlled room (50–55% humidity and 25 ± 2 °C) and under 12 h light: 12 h dark photoperiod at the Southern Laboratory Animal Facility, Prince of Songkla University, Thailand. All mice had free access to food and water. All experiments were approved and guided by the Animal Ethics Committee of the Prince of Songkla University, Thailand (Project license number: MOE 0521.11/1555, Ref.68/2018).

The mice were divided into six groups (n = 5–6 in each group): a normal control, a constipation control, a positive control, and three treatments of SPE. The normal and constipation control groups were supplemented with 0.2 mL of distilled water. The positive control group was supplemented with 0.2 mL of 500 mg lactulose /kg of body weight, and the treatment groups were supplemented with 0.2 mL of SPE at 100, 500, and 1000 mg/kg of body weight. Lactulose and SPE were administered daily by oral gavage for two weeks. To prepare SPE and lactulose solutions for daily administration, SPE powder was freshly dissolved in distilled water. In all mice, except mice in the normal control group, constipation was induced by injection of 5 mg/kg loperamide (Lop) on day 12, day 13, and day 14 [34,36]. The body weight of each mouse was recorded every day. 

On day 14, fecal pellets were collected for 4 h and then weighed and dried to calculate fecal water content. Gastrointestinal transit was also measured. The mice were anesthetized with 70 mg/kg thiopental sodium, and the small intestine, caecum, and colon were collected and dissected to study upper gut transit, colonic motility patterns, colonic smooth muscle contractility, epithelial transport in distal colon, and the composition of microbiota in cecal contents.

### 2.3. Measurement of Gastrointestinal Transit

To evaluate total gut transit time, mice were given a 0.1 mL Evans blue marker meal containing 5% Evans blue in 1.5% methylcellulose, and the time of the first blue pellet expulsion was recorded. A 3 mm glass bead was inserted into the colon (approximately 2 cm) using a plastic tip lubricated with petroleum jelly, and then the time to bead expulsion was recorded to observe the distal colonic transit time. For small intestinal transit, mice were gavage fed a 0.3 mL charcoal meal containing 10% *w*/*v* charcoal in 5% *w*/*v* gum arabic at 30 min before euthanasia. The euthanized mice were dissected, and transit (%) was calculated from the following equation [37]:

$$\mathrm{Small}\;\mathrm{intestinal}\;\mathrm{transit}\;\left(\%\right)=\frac{\mathrm{the}\;\mathrm{distance}\;\mathrm{of}\;\mathrm{charcoal}\;\mathrm{meal}}{\mathrm{total}\;\mathrm{length}\;\mathrm{of}\;\mathrm{the}\;\mathrm{small}\;\mathrm{intestine}}\times 100$$

### 2.4. Colonic Motility Pattern

After dissection, the whole colon with natural fecal pellets was collected and placed in ice-cold Krebs solution (pH 7.4 with an osmolality of 289–292 mmol/kg H_2_O) in an organ bath with a Gastrointestinal Motility Monitor (GIMM) (Catamount Research and Development, St. Albans, VT, USA) and then continuously perfused at 10 mL/min with fresh oxygenated Krebs solution. The colon was allowed to equilibrate for 30 min in Krebs solution at 37 °C. The movement of fecal pellets was recorded using a video camera above the chamber, and then the images from each individual run were analyzed, and we constructed the spatiotemporal maps of motility using GIMM software [20]. The contraction patterns comprised propagating contractions and non-propagating contractions. The total number of spontaneous contractions was defined as the sum of propagating and non-propagating contractions.

### 2.5. Colonic Smooth Muscle Contractility

To observe colonic smooth muscle contractility, the colon was first cleared of luminal content, and 1 cm colonic segments of proximal and distal colon were used and suspended in the direction of longitudinal smooth muscle fibers in a 10 mL organ bath containing oxygenated Krebs solution at 37 °C. To stimulate contraction, carbachol (Tocris Bioscience, Bristol, UK) was added to the Krebs solution in the organ bath in a cumulative fashion. The concentrations of carbachol progressed from 0.1 to 1 to 10 μM, without washing between increments. The amplitude of contraction (g) and frequency of contraction (times/min) were recorded with the PowerLab^®^ System (AD Instruments, New South Wales, Australia) and analyzed with LabChart7 program software [20,37].

### 2.6. Transepithelial Transport of Electrolytes across Cell Membranes in Distal Colon

To observe the transport of Na^+^ and Cl^−^ across the epithelial cell membrane, 1 cm of distal colon tissue was opened and oriented as a flat sheet on an Ussing slider, which was placed in an Ussing chamber (Physiologic Instruments, San Diego, CA, USA) containing Krebs solution at 37 °C [21]. Carbogen was also included in this system to maintain the buffer at the physiological pH of 7.4 during the experiment. After that, transepithelial voltage (V_t_) was recorded for 30 min as an equilibration period by injection of external current pulses (3 µA). To investigate Na^+^ absorption by distal colon, 10 µM amiloride was added to the chamber at the apical membrane to inhibit Na^+^ absorption by the epithelial sodium channel (ENaC), and the change in V_t_ was then recorded for 10 min. Cl^−^ secretion of Ca^2+^-activated Cl^−^ channels (CaCC) was then induced by adding 100 µM of carbachol to the chamber at the basolateral membrane, and the change in V_t_ was again recorded for 10 min. Cl^−^ secretion of the cystic fibrosis transmembrane conductance regulator (CFTR) was then induced by adding 10 µM forskolin at the basolateral membrane, and the changes in V_t_ were recorded for 10 min. Following Ohm’s law, the transepithelial potential difference (V_te_), transepithelial resistance (R_te_), and equivalent short-circuit current (I_sc_) were calculated to represent the transepithelial transport of electrolytes in the collected distal colon [38,39].

### 2.7. Composition of Colonic Microbiota Analyses

Bacterial DNA of all samples were extracted from collected cecal content [40]. To amplify and detect bacterial 16S rRNA genes, qPCR was performed using LineGene 9600 Plus System (BIOER, Hangzhou, China) and SensiFAST™ SYBR^®^ No-ROX Kit (Bioline). The following primer sets were used: FW 5′-CGATGAGTGCTAGGTGTTGGA-3′ and RV 5′-CAAGATGTCAAGACCTGGTAAG-3′ for total bacteria, LM26 5′-GATTCTGGCTCAGGATGAACGC-3′ and Bif228 5′-CTGATAGGACGCGACCCCAT-3′ for Bifidobacteria, FW 5′-CGATGAGTGCTAGGTGTTGGA-3′ and RV 5′-CAAGATGTCAAGACCTGGTAAG-3′ for Lactobacilli, F-ent 5′- ATGGCTGTCGTCAGCTCGT-3′ and R-ent 5′-CCTACTTCTTTTGCAACCCACTC-3′ for Enterobacteriaceae, and ECF 5′-AGAAATTCCAAACGAACTTG-3′ and ECR 5′-CAGTGCTCTACCTCCATCATT-3′ for Enterococci [41,42,43,44,45]. The following thermal cycling condition was used for all amplifications: 3 min at 95 °C, followed by 40 cycles of a two-step PCR reaction (5 s at 95 °C and 30 s at 60 °C) [40].

### 2.8. Statistical Analysis

All data are presented as means ± standard error (SE). The differences between groups were tested using one-way or two-way analysis of variance (ANOVA), followed by Bonferroni’s test at α = 0.05 using GraphPad Prism 5 (version 5.01). 

## 3. Results

### 3.1. Effect of SPE Pretreatment on Body Weight, Fecal Water Content, and Gut Transit in Constipated Mice

**Figure 1:**
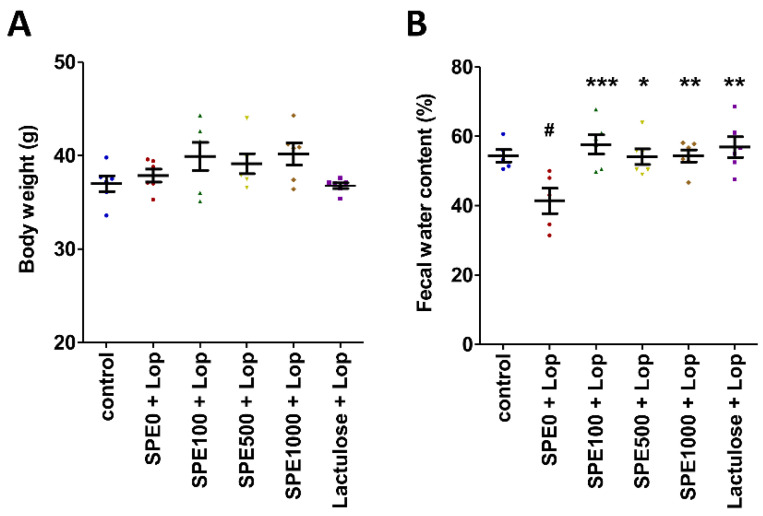
Effects of *Sargassum plagiophyllum* extract (SPE) pretreatment on body weight and fecal water content of constipated mice. (**A**) Body weight and (**B**) fecal water content of normal control group (control); constipation control group (SPE0 + Lop); 100, 500, and 1000 mg/kg SPE treatment groups (SPE100 + Lop, SPE500 + Lop, and SPE1000 + Lop, respectively); and positive control group (Lactulose + Lop). Symbols above the bars indicate significant differences from normal control or constipation control (# means *p* < 0.05 when compared with normal control group, and *, **, and *** mean *p* < 0.05, 0.01, and 0.001, respectively, when compared with constipation control group).

On day 14, the body weight of the mice in all treatment groups was not significantly different (Figure 1A, *p* > 0.05). Fecal water content was significantly lower in the constipation control group than in the normal control group (Figure 1B, *p* < 0.05). Fecal water content was significantly higher in the lactulose and SPE treatment groups than in the constipation control group (*p* < 0.05).

**Figure 2:**
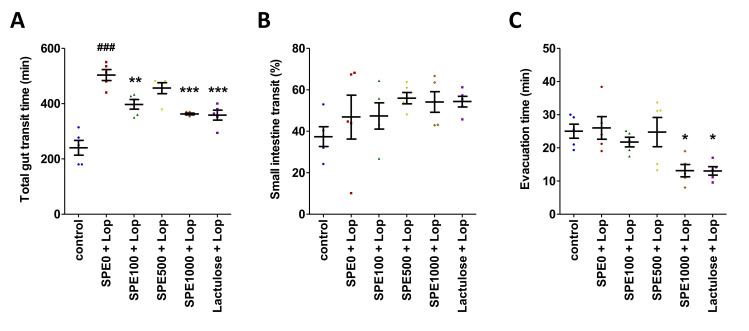
Effects of *Sargassum plagiophyllum* extract (SPE) pretreatment on gut transit of constipated mice. (**A**) Total gut transit time, (**B**) small intestine transit, and (**C**) evacuation time of normal control group (control); constipation control group (SPE0 + Lop); 100, 500, and 1000 mg/kg SPE treatment groups (SPE100 + Lop, SPE500 + Lop, and SPE1000 + Lop, respectively); and positive control group (Lactulose + Lop). Symbols above the bars indicate significant differences from normal control or constipation control (### means *p* < 0.001 when compared with the normal control group, and *, **, and *** mean *p* < 0.05, 0.01, and 0.001, respectively, when compared with the constipation control group).

The effects of SPE treatment on gut transit were determined using the total gut transit time, small intestinal transit time, and evacuation time (Figure 2). The total gut transit time in the constipation control group was 503.60 ± 19.78 min. The total gut transit time in the normal control group was significantly shorter at 240.20 ± 26.59 min (Figure 2A, *p* < 0.001). The total gut transit time was also shorter in all three SPE treatment groups, and it was the shortest in the 1000 mg/kg SPE group (*p* < 0.001). The small intestinal transit time was not significantly different among all groups (Figure 2B). The evacuation time was slightly longer in the constipation control group (26.01 ± 3.40 min) than in the normal control group (25.02 ± 2.13 min), but it was not significantly different (Figure 2C, *p* > 0.05). However, the evacuation times were significantly shorter in the positive control (Lactulose + Lop) group and the 1000 mg/kg SPE treatment group than in the constipation control group (*p* < 0.05). Our results suggest that SPE pretreatment could shorten total gut transit time and evacuation time.

### 3.2. Effect of SPE Pretreatment on Colonic Motility Pattern in Constipated Mice

**Figure 3:**
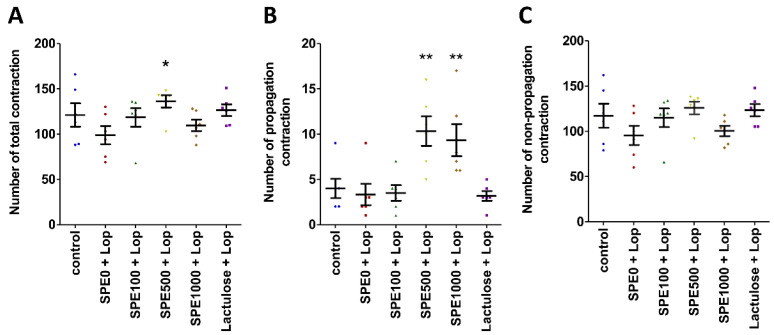
Effects of *Sargassum plagiophyllum* extract (SPE) pretreatment on the colonic motility pattern of constipated mice. (**A**) Number of total contractions, (**B**) number of propagation contractions, and (**C**) number of non-propagation contractions of normal control group (control); constipation control group (SPE0 + Lop); 100, 500, and 1000 mg/kg SPE treatment groups (SPE100 + Lop, SPE500 + Lop, and SPE1000 + Lop, respectively); and positive control group (Lactulose + Lop). Symbols above the bars indicate significant differences from constipation control (* and ** mean *p* < 0.05 and 0.01, respectively, when compared with the constipation control group).

The colonic motility pattern was investigated by determining the total number of contractions, the number of propagation contractions (peristalsis), and the number of non-propagation contractions (segmentation). Spatiotemporal maps were produced from an analysis of the contraction data using GIMM software (Figure 3). The total number of contractions was insignificantly higher in the normal control group than in the constipation control group (Figure 3A, *p* > 0.05), but the total number of contractions was significantly higher in the 500 mg/kg SPE treatment group than in the constipation control group (*p* < 0.05). Moreover, the number of propagation contractions was also significantly higher in the 500 and 1000 mg/kg SPE treatment groups than in the constipation control group (Figure 3B, *p* < 0.01). Non-propagation contractions were not significantly different among the groups (Figure 3C, *p* > 0.05).

### 3.3. Effect of SPE Pretreatment on Colonic Smooth Muscle Contractility in Constipated Mice

**Figure 4:**
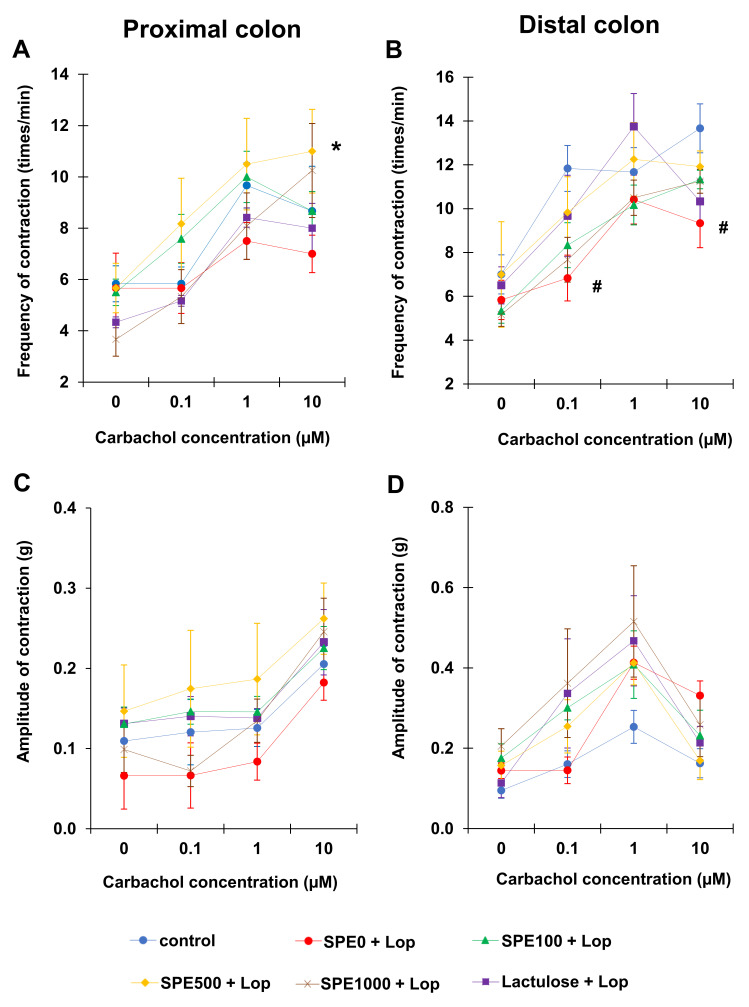
Effects of *Sargassum plagiophyllum* extract (SPE) pretreatment on colonic smooth muscle contractility of constipated mice. Frequency and amplitude of contractions of (**A**,**C**) proximal colon and (**B**,**D**) distal colon of normal control group (control); constipation control group (SPE0 + Lop); 100, 500, and 1000 mg/kg SPE treatment groups (SPE100 + Lop, SPE500 + Lop, and SPE1000 + Lop, respectively); and positive control group (Lactulose + Lop). Symbols indicate significant differences from normal control or constipation control (# means *p* < 0.05 when compared with the normal control group, and * means *p* < 0.05 when compared with the constipation control group).

The amplitude and frequency of the contractions of the longitudinal smooth muscle fibers of the proximal and distal colon were observed to investigate the colonic smooth muscle contractility (Figure 4). The results revealed that the contractions of both the proximal and distal colon tended to be more frequent in the positive control (Lactulose + Lop) and SPE treatment groups. After adding 10 μM of carbachol, proximal colonic contractions occurred significantly less frequently in the constipation control (SPE0 + Lop) group (7.00 ± 0.73 times/min) than in the 500 mg/kg SPE treatment group (11.00 ± 1.63 times/min) (Figure 4A, *p* < 0.05). Distal colonic contractions were also significantly less frequent in the constipation control group (SPE0 + Lop) (9.33 ± 1.54 times/min) than in the normal control group (13.67 ± 1.12 times/min) (Figure 4B, *p* < 0.05). Even at 0.1 μM, contractions in the distal colon were significantly less frequent (6.83 ± 0.95 times/min) in the constipation control group than in the normal control group (11.83 ± 1.05 times/min) (*p* < 0.05). 

The amplitude of the proximal colonic contractions showed a similar trend in all groups in that the amplitude of the contractions was highest at 10 µM of carbachol (Figure 4C). The amplitude of the proximal colonic contractions was lower in the constipation control group than in the normal control and positive control groups, as well as the 100, 500, and 1000 mg/kg SPE treatment groups, but there was no significant difference among all groups at all concentrations of carbachol (*p* > 0.05). The amplitude of the distal colonic contractions was highest at 1 µM of carbachol, but there was, again, no significant difference among all groups at all concentrations of carbachol (Figure 4D, *p* > 0.05).

### 3.4. Effect of SPE Pretreatment on Transport of Electrolytes across Cell Membranes in Distal Colon of Constipated Mice

**Figure 5:**
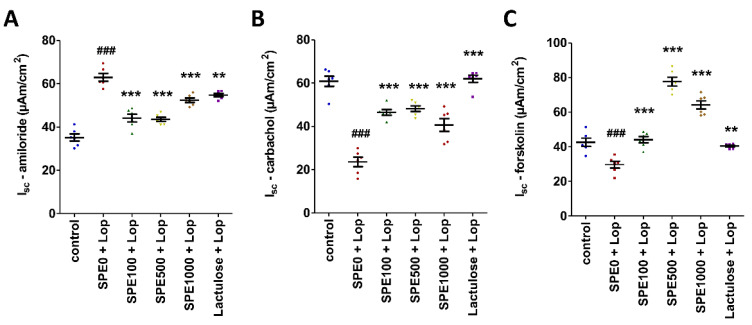
Effects of *Sargassum plagiophyllum* extract (SPE) pretreatment on transport of electrolytes across cell membranes of constipated mice. The charts show short-circuit current (I_sc_) responses to (**A**) amiloride, (**B**) carbachol, and (**C**) forskolin of distal colon in normal control group (control); constipation control group (SPE0 + Lop); 100, 500, and 1000 mg/kg SPE treatment groups (SPE100 + Lop, SPE500 + Lop, and SPE1000 + Lop, respectively); and positive control group (Lactulose + Lop). Symbols above the bars indicate significant differences from normal control or constipation control (### mean *p* < 0.001, respectively, when compared with the normal control group, and ** and *** mean *p* < 0.01 and 0.001, respectively, when compared with the constipation control group).

The basal transport values (V_te_, R_te_, and I_sc_) of the distal colon were not significantly different among the groups (Table 1). However, these values did show significant differences when the distal colon was exposed to amiloride, carbachol, and forskolin. The amiloride-induced I_sc_ of the distal colon in the constipation control group (62.95 ± 1.77 µAm/cm^2^) was significantly higher than the amiloride-induced I_sc_ of the distal colon in the normal control (*p* < 0.001), positive control (*p* < 0.01), and SPE treatment (*p* < 0.001) groups (Figure 5A). In contrast, the carbachol-induced I_sc_ of the distal colon in the constipation control group (19.46 ± 3.13 µAm/cm^2^) was significantly lower than the carbachol-induced I_sc_ of the distal colon in the normal control, positive control, and SPE treatment groups (Figure 5B, *p* < 0.001). The forskolin-induced I_sc_ of the distal colon was also lower in the constipated control group (29.65 ± 1.92 µAm/cm^2^) than in the normal control (*p* < 0.001), positive control (*p* < 0.01), and SPE treatment (*p* < 0.001) groups (Figure 5C).

### 3.5. Effect of SPE Pretreatment on Composition of Gut Microbiota in Constipate Mice

**Figure 6:**
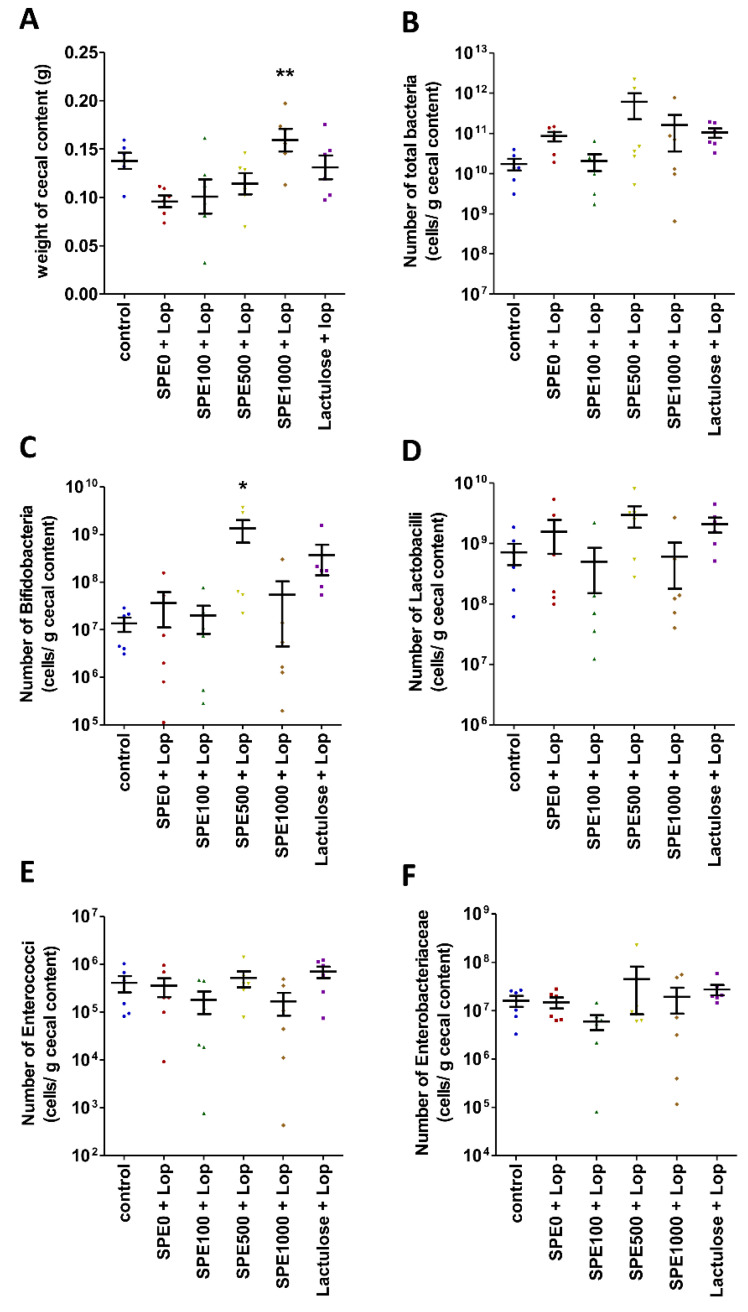
Effects of *Sargassum plagiophyllum* extract (SPE) pretreatment on gut microbiota in constipated mice. (**A**) Weight of cecal contents and number of (**B**) total bacteria, (**C**) Bifidobacteria, (**D**) Lactobacilli, (**E**) Enterococci, and (**F**) Enterobacteriaceae in cecal contents of normal control group (control); constipation control group (SPE0 + Lop); 100, 500, and 1000 mg/kg SPE treatment groups (SPE100 + Lop, SPE500 + Lop, and SPE1000 + Lop, respectively); and positive control group (Lactulose + Lop). Symbols above the bars indicate significant differences from constipation control (* and ** mean *p* < 0.05 and 0.01, respectively, when compared with the constipation control group).

Cecal contents were collected and weighed to estimate the numbers of total bacteria, Bifidobacteria, Lactobacilli, Enterobacteriaceae, and Enterococci in the cecum of mice from every control group and all SPE treatment groups. The cecal content weight of the mice in the 1000 mg/kg SPE treatment group (0.1584 ± 0.0117 g) was significantly higher than the cecal content weight of the mice in the constipation control group (0.0960 ± 0.0061 g) (Figure 6A, *p* < 0.01), but the numbers of total bacteria were not significantly different among groups (Figure 6B, *p* > 0.05). The number of Bifidobacteria was not significantly different between the constipation control and the normal control groups (*p* > 0.05), but it was significantly higher in the 500 mg/kg SPE treatment group (1.33 ± 0.66 × 10^9^ cells/g cecal content) than in the constipation control group (6.78 ± 3.42 × 10^7^ cells/g cecal content) (Figure 6C, *p* < 0.05). The numbers of Lactobacilli, Enterobacteriaceae, and Enterococcus were not significantly different among groups (Figure 6D–F, *p* > 0.05). Our results suggest that pretreatment with 500 mg/kg SPE could modulate the composition of bacteria, especially Bifidobacteria, in the cecum of constipated mice.

## 4. Discussion

Our results suggest that SPE pretreatment increased the frequency of contractions in the colonic smooth muscle and effectively increased both the propagation contractions and the total contractions of the colon in constipated mice. Moreover, the frequency of the contractions of the constipation control mice was lower than that of the others; therefore, the total gut transit time of the constipation control mice was longer than that of the normal control and SPE pretreatment groups. This indicates that SPE is capable of preventing constipation by enhancing colonic contraction and reducing the gut transit time and evacuation time. The results of this study are consistent with the results of a previous study of the marine algae *Ulva* (*Enteromorpha*), which indicated that dried *Ulva* enhanced colonic contraction and reduced gut transit time in constipated mice [46].

A recent study found polysaccharides, such as alginates, laminarins, and fucoidans, in *Sargassum* [47]. These polysaccharides have been used as substrates for the fermentation and production of short-chain fatty acids (SCFAs) by gut microbiota [48]. The present study showed that the Bifidobacteria population in the cecal contents of constipated mice was significantly increased in mice pretreated with 500 mg/kg SPE. Bifidobacteria are beneficial microorganisms that stimulate the growth of butyrate-producing bacteria, such as *Faecalibacterium*, *Eubacterium,* and *Roseburia* [49]. Acetate, propionate, and butyrate have been shown to interact with the free fatty acid receptors 2 and 3 (FFA2 and FFA3) of enterochromaffin cells (ECs) to induce serotonin (5-HT) release and trigger peristalsis [50]. Our results show that the frequency of the total colonic contractions was higher and the total gut transit time was shorter in the SPE treatment groups than in the constipation control group. Pretreatment with SPE was therefore able to prevent constipation by enhancing colonic contractions and reducing the gut transit time and evacuation time. Therefore, SPE pretreatment prevented constipation in the loperamide-induced mice by promoting the beneficial bacteria that might enhance the butyrate production, which leads to increased colonic contractility. In this study, we observed four selected bacteria that were reported to be involved with constipation. For further studies, we suggest that 16S rRNA gene sequence analysis should be performed to observe the changes in the microbiota of SPE-treated mice.

Furthermore, the results of this study also suggest that SPE pretreatment increases fecal water content in constipated mice. Our results are consistent with those of previous studies that revealed a reduction in fecal water content and the secretion of water in the distal colon of constipated rats [51]. The feces of mice supplemented with lactulose and SPE in this study contained more water than the feces of mice in the constipation control group. This result supports the findings of studies of the marine algae *Ulva* and *Chondrus*. These algae induced the secretion of water into the colon and increased fecal water content [46,52,53]. Moreover, lactulose was also found to increase fecal water content by absorption [54]. 

The study of electrolyte transport across the epithelial cell membrane of the distal colon showed that the basal transepithelial potential difference (V_te_), transepithelial resistance (R_te_), and equivalent short-circuit current (I_sc_) were not significantly different among all groups. This result indicates that pretreatment with SPE did not affect the colonic tissue or the ion channels [39]. However, the functioning of the ion channels in the distal colon of constipated mice treated with SPE changed. Cl^−^ secretion increased, and Na^+^ absorption was inhibited in SPE-pretreated mice. These changes increased the fecal water content in these groups compared with the constipation control group. The increased Cl^−^ secretion was confirmed by the increased I_sc_ induced by carbachol in the distal colon of SPE-supplemented mice. SPE induced an influx of Ca^2+^ into gut epithelial cells, which activated the CaCC [55]. Moreover, the forskolin-induced I_sc_ was also higher in the SPE pretreatment groups, indicating that cAMP increased in the cell and then activated CFTR and increased Cl^−^ secretion [56]. *Sargassum* has been shown to contain flavonoids [57], which increase cyclic adenosine monophosphate (cAMP) in gut epithelial cells and then induce the release of Ca^2+^ from the endoplasmic reticulum to the cytosol via protein kinase A (PKA) [54,58]. Recent studies revealed that increased cellular Ca^2+^ levels are not only important for the activation of CaCC but that they also activate CFTR via the PI3K/Akt pathway [56,59]. Therefore, SPE pretreatment might increase fecal water content by increasing cellular Ca^2+^ levels to induce Cl^−^ secretion in the colonic lumen.

High cellular Ca^2+^ levels also inhibited Na^+^ absorption and reduced water absorption in the colon [60]. In the present study, the amiloride-induced I_sc_ of the distal colon of mice treated with SPE decreased. This result indicates that Na^+^ absorption in the distal colon reduced, and fecal water content therefore increased. In a recent study, it was found that goblet cell numbers on the villi of ileum increased in constipated mice pretreated with SPE [34]. This finding implied that mucus secretion in the ileum might also have increased, which supports our finding that fecal water content increased in constipated mice supplemented with SPE.

In conclusion, SPE is a natural supplement that enhances colonic contractility and increases the numbers of Bifidobacteria. Pretreatment with SPE reduced the gut transit time and the evacuation time of constipated mice. SPE also increased the secretion of Cl^−^ and reduced Na^+^ absorption in the distal colon, leading to increased fecal water content. Therefore, SPE was able to prevent constipation.

## Figures and Tables

**Table 1:** Transepithelial potential difference (V_te_), transepithelial resistance (R_te_), and equivalent short-circuit current (I_sc_) of distal colonic epithelium membrane of normal control, constipation control, positive control, and SPE-pretreated mice.

Treatment	V_te_ (V)	R_te_ (Ω.cm^2^)	I_sc_ (µA/cm^2^)
Control	8.33 ± 1.89	71.57 ± 7.28	119.74 ± 23.50
0 mg/kg SPE + Loperamide	8.22 ± 1.63	57.23 ± 7.38	147.51 ± 25.31
100 mg/kg SPE + Loperamide	8.53 ± 1.93	59.47 ± 6.55	136.40 ± 22.98
500 mg/kg SPE + Loperamide	6.51 ± 1.18	58.68 ± 5.45	108.59 ± 14.58
1000 mg/kg SPE + Loperamide	8.58 ± 1.35	72.18 ± 10.24	121.79 ± 12.04
500mg/kg Lactulose + Loperamide	5.37 ± 0.88	57.72 ± 8.19	100.08 ± 21.06

## Data Availability

The data supporting the research for this study are available within the manuscript.
